# Exercise prevents fatal stress-induced myocardial injury in obese mice

**DOI:** 10.3389/fendo.2023.1223423

**Published:** 2023-08-29

**Authors:** Yaoshan Dun, Zihang Hu, Baiyang You, Yang Du, Lingfang Zeng, Yue Zhao, Yuan Liu, Shaoping Wu, Ni Cui, Fan Yang, Suixin Liu

**Affiliations:** ^1^Division of Cardiac Rehabilitation, Department of Physical Medicine & Rehabilitation, Xiangya Hospital of Central South University, Changsha, China; ^2^School of Cardiovascular and Metabolic Medicine & Sciences, King’s College London British Heart Foundation Centre of Excellence, Faculty of Life Sciences and Medicine, King’s College London, London, United Kingdom; ^3^National Clinical Research Center for Geriatric Disorders, Xiangya Hospital of Central South University, Changsha, China; ^4^Division of Preventive Cardiology, Department of Cardiovascular Medicine, Mayo Clinic, Rochester, MN, United States; ^5^Department of Neurology, Xiangya Hospital of Central South University, Changsha, China; ^6^School of Cardiovascular and Metabolic Medicine & Sciences, Faculty of Life Sciences and Medicine, King’s College London, London, United Kingdom

**Keywords:** obesity, aerobic exercise, cardiac function, myocardial injury, mitochondrial quality control

## Abstract

**Introduction:**

This study aimed to explore whether aerobic exercise (AE) can prevent fatal stress-induced myocardial injury.

**Methods:**

Thirty C57BL/6J mice were divided into either a normal diet, high-fat diet, or high-fat diet plus AE (n=10 per group). The AE protocol consisted of eight weeks of swimming. At the end of the diet and AE interventions, the mice were stimulated with fatal stress caused by exhaustive exercise (forced weight-loaded swimming until exhaustion), after which cardiac function was evaluated using echocardiography, myocardial ultrastructure was examined using transmission electron microscopy, and myocardial apoptosis was assessed using western blotting and TUNEL. Mitophagy, mitochondrial biogenesis and dynamics, and activation of the macrophage migration inhibitor factor (MIF)/AMP-activated protein kinase (AMPK) pathway were evaluated using quantitative PCR and western blotting. Obesity phenotypes were assessed once per week.

**Results:**

AE reversed high-fat diet-induced obesity as evidenced by reductions in body weight and visceral fat compared to obese mice without AE. Obesity exacerbated fatal stress-induced myocardial damage, as demonstrated by impaired left ventricular ejection fraction and myocardial structure. The apoptotic rate was also elevated upon fatal stress, and AE ameliorated this damage. Obesity suppressed mitophagy, mitochondrial fission and fusion, and mitochondrial biogenesis, and these effects were accompanied by suppression of the MIF/AMPK pathway in the myocardium of mice subjected to fatal stress. AE alleviated or reversed these effects.

**Conclusion:**

This study provides evidence that AE ameliorated fatal stress-induced myocardial injury in obese mice. The cardioprotective effect of AE in obese mice might be attributed to improved mitochondrial quality.

## Highlights

1. Obesity exacerbated the fatal stress-induced cardiac dysfunction and myocardial injury.
2. Aerobic exercise preconditioning mitigated fatal stress-induced myocardial damage.
3. The study provides more evidence supporting the use of exercise as a preventive measure for cardiovascular events in individuals with obesity.

## Introduction

Adverse cardiovascular events, such as cardiovascular death and heart attack, remain the leading causes of death worldwide (1). Obesity has escalated globally in recent years, and individuals with obesity exhibit a higher risk of adverse cardiovascular events than individuals of normal weight (2). Altered energy homeostasis in individuals with obesity results in a significant myocardial burden (3), blunted stress response (4), and increased susceptibility to severe injury in patients with myocardial damage (5). Thus, exploring ways to prevent adverse cardiovascular events in obesity is clinically significant.

A growing number of studies have explored the impacts of interventions in alleviating myocardial injury in animal models, generally through permanent ligation of the left anterior descending coronary artery (6) and ischemia-reperfusion injury (7), which mimic heart attack and revascularization-related damage, respectively. Limited studies, to our knowledge, have explored strategies to prevent myocardial injury from extreme stresses, such as heavy lifting and straining. However, extreme stresses are the common triggers of adverse cardiovascular events (8, 9), and thus warrant further research.

Our recent clinical study showed that exercise-based cardiac rehabilitation significantly prevented cardiovascular death and recurrent heart attack (10). In addition, recent preclinical studies from our team investigated drug (11, 12) and non-drug (11–13) preconditioning strategies to ameliorate muscle damage caused by fatal stress induced by exhaustive exercise in non-obese mice. We found that 8–12 weeks of aerobic exercise (AE) significantly ameliorated fatal stress-induced skeletal (11) and myocardial damage in non-obese mice (12, 13). This improvement was accompanied by the enhancement of mitochondrial quality control (MQC) (11, 12), and the reduction of free radical levels, as evidenced by the increase of superoxide dismutase and decrease in malondialdehyde (11). Additionally, increasing evidence suggests that nuclear factor erythroid 2-related factor 2 (Nrf2), a master regulator of antioxidant defenses, and its downstream targets play a crucial role in how oxidative stress mediates the beneficial effects of exercise (14, 15). However, the effect of AE preconditioning on the prevention and alleviation of exhaustive exercise-induced myocardial injury in obese mice remains unknown.

Mitochondria are responsive to exercise in muscle (16), and their quality is closely related to organ stress responses (17). At the organelle level, MQC stands for the collaboration between mitophagy, mitochondrial dynamics, and biogenesis (18). A recent study by Sliter et al. demonstrated that suppressed mitophagy aggravated acute myocardial injury in mice (19). Additionally, in our experimental study with mice, we observed that AE rectified skeletal muscle atrophy induced by a high-fat diet (HFD) and impairments in mitochondrial oxygen consumption rate in myotubes (20). These studies suggest that MQC may be involved in AE-induced cardio protection.

AMP-activated protein kinase (AMPK) is a regulator of mitochondrial biogenesis and mitophagy (21). Our recent studies have reported that AE increases AMPK activity in the skeletal muscle (20) and livers (22) of obese mice. In addition, a study by Ma et al. suggested that impaired macrophage migration inhibitory factor (MIF)/AMPK pathway activation is associated with myocardial infarct size in mice (23). Moreover, a study by Moon et al. suggested that MIF may play a role in the antidepressant effects of exercise (24).

In this study, we aimed to explore whether 8-week AE preconditioning can prevent fatal stress-induced myocardial injury and preliminarily investigated the roles of mitophagy, mitochondrial dynamics and biogenesis, and the MIF/AMPK pathway in AE-mediated cardioprotection in obese mice.

## Materials and methods

### Animals and study design

**Figure 1:**
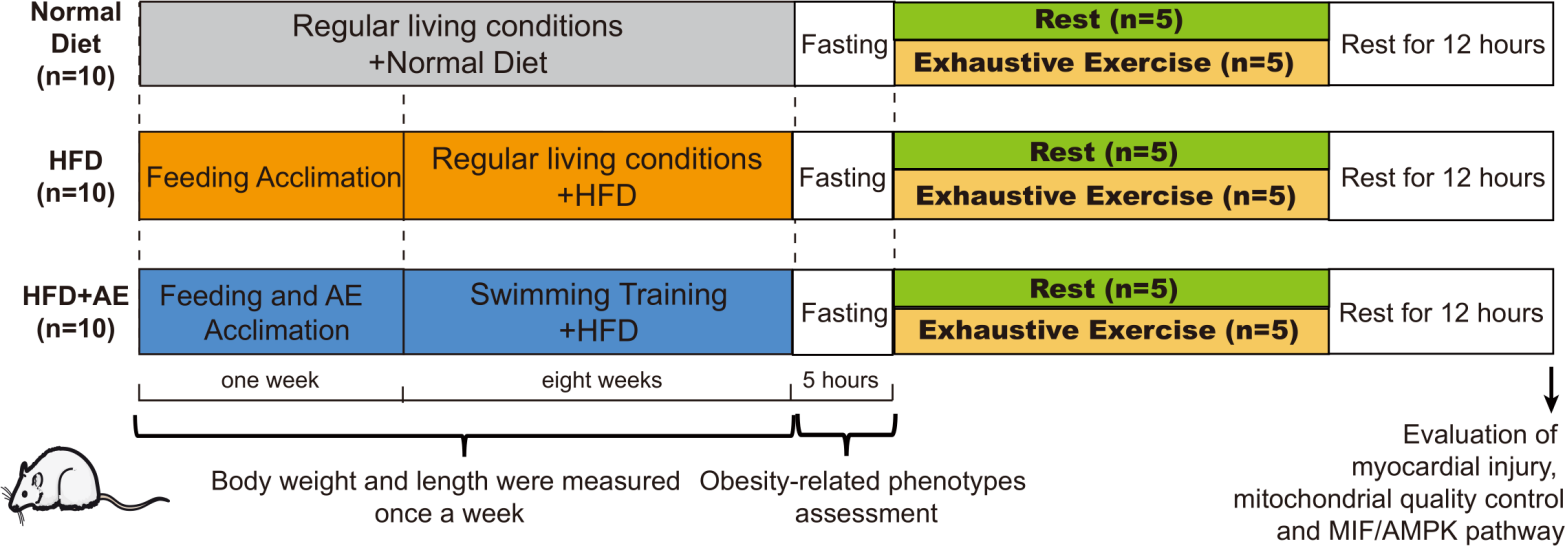
A schematic of the experimental design. Thirty C57BL/6J male mice were randomly divided into a normal diet, high-fat diet (HFD), or HFD plus aerobic exercise (AE) (n=10 per group). Mice in the HFD plus AE group swam for 60 min per day, 5 days per week, for 8 weeks. Obesity phenotypes were assessed once per week (n=10 per group). After eight weeks of intervention, obesity-related phenotypes, such as fasting blood glucose (n=10 per group) and insulin resistance levels (n=5 per group), were evaluated. Half of the mice in each group were randomly chosen to perform exhaustive exercise (forced weight-loaded swimming until exhaustion) to induce fatal stress (n=5 per group); the other half of the mice were allowed to rest without further intervention (n=5 per group). Prior to sacrifice, all mice were allowed 12 h of rest. Serum creatine kinase (CK) levels were detected, and the myocardial ultrastructure was determined using transmission electron microscopy (n=5 per group). Myocardial apoptosis was assessed using western blotting and TUNEL staining (n=5 per group). Biomarkers of mitochondrial quality control in the myocardium were evaluated using western blotting (n=5 per group).

We obtained eight-week-old male C57BL/6J mice from the Laboratory Animal Center at Xiangya Medical School in Changsha, China. The mice were kept in standard conditions with a temperature of 22 ± 2°C, humidity levels of 45–55%, and a regular 12-hour light-dark cycle. The padding was renewed on alternate days. Following a one-week feeding adaptation period, the mice were divided into three groups receiving different diets (n=10 per group) based on the different interventions they were assigned to receive. These interventions included a normal diet, a high-fat diet (HFD), and an HFD plus AE preconditioning. The normal diet group was fed standard chow consisting of 15% fat, 20% protein, and 65% carbohydrates. Mice in the HFD groups were fed an HFD containing 45% fat, 20% protein, and 35% carbohydrates (FBSH BIO-PHARMACEUTICAL, Shanghai, China) for eight weeks. Mice had free access to food and water. Mice subjected to AE preconditioning followed by our AE training protocol described in the section *Aerobic Exercise Training Protocol* below. The experimental design is illustrated in **Figure 1**. All protocols were approved by the Experimental Animal Welfare Ethics Committee of Central South University (approval number: CSU-2022-0282) and were in line with the Medicine Institute of Health’s guidelines for the use of live animals. All animal experiments complied with the ARRIVE guidelines.

### Aerobic exercise training protocol

The mouse AE training protocol followed the current guidelines for mice exercise and training (25) and has been reported in our previous study (12). Briefly, AE was conducted for 60 min per day, five days per week for eight weeks. Acclimatization training was also applied: one week before formal AE, the mice started with 10 min of swimming training on the first day, followed by daily increases in 10 min increments until they reached 60 min per day. After acclimatization, mice at nine weeks of age were forced to swim in a Morris water maze for 60 min per day with a wooden rod tied to its tail. All of the exercise training sessions were scheduled between 9 a.m. and 2 p.m.

### Exhaustive exercise-induced fatal stress

Exhaustive exercise (EE) has been shown to induce fatal stress in mice. We previously successfully used this method in both non-obese (12) and obese mice (20). Briefly, after the 8-week intervention, 5 mice were randomly selected from each group to perform exhaustive exercise and forced weight-loaded swimming until exhaustion. The criteria for exhaustion were defined as sinking to the bottom of the Morris water maze and failing to hedge to the water surface to breathe for seven seconds. To force the mice to swim, a lead sheath load (five percent of each mouse’s weight) was bound to their tails. A session of adaptive weight-loaded swimming with an added load on the tail was provided to the mice before the day of the exhaustive exercise.

### Exercise capacity and cardiac function assessment

The maximal swimming time was noted as an indicator of exercise capacity (n=15, five per group) (11, 12). The echocardiography of the mice was performed prior to their sacrifice. M-mode echocardiography (Mindray Inc., Nanjing, China) under anesthetization was used to obtain the left ventricular ejection fraction (LVEF) and left ventricular internal dimensions. An experienced investigator, who was not aware of the treatment allocation, conducted all of the analyses. The formula V = [7.0/(2.4+D)]×D³ was utilized for calculating the left ventricular end-diastolic volume (LVEDV) and left ventricular end-systolic volume (LVESV). Subsequently, the LVEF was calculated using the formula: LVEF (%) = (LVEDV-LVESV)/LVEDV×100%.

### Assessment of obesity-related phenotypes

Body weight was measured and recorded weekly. Visceral fat weight, visceral fat mass/body weight ratio, and fasting blood glucose (FBG) levels were assessed at the end of the intervention in all mice (n=30, ten per group). We isolated and weighted visceral adipose tissues, consisting of perirenal, mesenteric, and unilateral epididymal adipose (n=30, ten per group). The visceral adipose was then fixed and embedded in paraffin for hematoxylin and eosin staining (n=15, five per group). The visceral adipocyte area was calculated by ImageJ as described previously (26). For FBG, blood was collected from the tail vein after 5 h of fasting and assayed for glucose content by Accu-Chek glucose meter (Roche Diagnostics, USA) (n=30, ten per group). Mice had free access to water during the fasting period.

Serum insulin levels were evaluated at the end of the intervention in mice without a fatal stress stimulus (n=15, five per group). Serum was obtained by removing the eyeball after euthanasia via pentobarbital sodium injection (150 mg/kg body weight). We used an enzyme-linked immunosorbent assay (ELISA) to measure insulin concentration (CSB-E05071m; Wuhan, China). Insulin resistance was estimated via the homeostasis model assessment for insulin resistance (HOMA-IR): HOMA-IR = FBG (mmol/L) × fasting insulin (mU/L)/22.5.

### Myocardial injury assessment

To verify fatal stress-induced myocardial injury, serum creatine kinase (CK) levels and myocardial ultrastructure were evaluated and compared between mice with (n=5 per group) and without (n=5 per group) fatal stress stimulus. The difference in response to fatal stress between normal-diet and HFD mice was also assessed. We used a commercial kit to measure serum CK levels (A032; Nanjing Jiancheng, China). The ultrastructure of the left ventricular myocardium was assessed using transmission electron microscopy (12).

To assess the degree of myocardial damage, we examined the activation of the apoptosis pathway using western blotting and by measuring the apoptotic rate using terminal deoxynucleotidyl transferase dUTP nick end labelling (TUNEL) in left ventricular myocardium (n=5 for each group). TUNEL assays were performed using a commercial kit (40306ES50, Yeasen, Shanghai, China) following the manufacturer’s instructions. Apoptosis in the myocardium was observed under a fluorescence microscope (BA410T, Motic) at 400x magnification, and myocardial cells with green fluorescence staining were considered TUNEL-positive. TUNEL-positive cells were counted to assess the proportion of apoptotic cells using image analysis software (ImageJ, version 1.49, USA).

### Western blotting

The expression of biomarkers of mitophagy, mitochondrial biogenesis, and mitochondrial fission and fusion (the components of MQC), as well as MIF and AMPK proteins, were determined using western blotting. Left ventricular myocardium protein extracts were obtained upon homogenization of tissues in RIPA lysis buffer (89901, Thermo Scientific, USA). After centrifuging the homogenates, the supernatants were analyzed as previously described (20). Polyvinylidene fluoride membranes with transferred proteins were blocked with 5% bovine serum albumin in phosphate-buffered saline plus 0.1% Triton X-100 (PBST) buffer before incubating with primary antibodies overnight at 4°C. The primary antibodies used were MIF (20415-1-AP, Proteintech, USA), AMPK (66536-1-Ig, Proteintech, USA), phosphorylated AMPK (pAMPK, ab23875, Abcam, UK), PTEN-induced kinase 1 (PINK1, 23274-1-AP, Proteintech, USA), microtubule-associated protein 1 light chain 3 (LC3, 14600-1-AP, Proteintech, USA), p62 (18420-1-AP, Proteintech, USA), mitofusin 1 (MFN1, 13798-1-AP, Proteintech, USA), DRP1 (12957-1-AP, Proteintech, USA), peroxisome proliferator-activated receptor gamma coactivator 1-alpha (PGC-1α, 66369-1-Ig, Proteintech, USA), Sirtuin 1 (SIRT1, 13161-1-AP, Proteintech, USA), citrate synthase (CS, 16131-1-AP, Proteintech, USA), GAPDH (10494-1-AP, Proteintech, USA), cytochrome c (10993-1-AP, Proteintech, USA), B-cell lymphoma 2 (BCL-2, ab182858, Abcam, UK), and BCL-2-associated X protein (BAX, ab32503, Abcam, UK). Goat anti-Rabbit IgG (H+L) (AWS0002, Abiowell, CHN) was used as a secondary antibody, and the immunoblots were visualized and documented using an enhanced chemiluminescence substrate. Myocardial proteins were observed and compared with a common protein standard loaded onto the gel using Quantity ONE.

### Reverse transcription and quantitative PCR

We extracted left ventricular myocardium mRNA using TRIzol reagent (15596026, Thermo, America). The concentration of mRNA was measured spectrophotometrically and RNA purity was assessed using the 260/280 ratio. RNA reverse-transcription, cDNA amplification, and PCR quantification were done by the PikoReal™ Real-Time PCR System. MIF gene expression was quantified using the 2^-ΔΔCt^ method, with GAPDH and β-actin as housekeeping genes. The used PCR primer sequences are listed in the **Table 1**.

**Table 1:** Sequence of primers.

Genes	Species	Sequence
MIF	Mouse	F- CATGACTTTTAGCGGCACGAACGA R- ACCACCGATCTTGCCGATGCTG
GAPDH	Mouse	F- GCACCACCAACTGCTTAG R- GGATGCAGGGATGATGTTC
β-actin	Mouse	F- GTGCTATGTTGCTCTAGACTTCG R- ATGCCACAGGATTCCATACC

### Sample size

Three types of interventions were introduced into mice: control, HFD, and HFD plus aerobic exercise, each contained 2 groups of control and exhaustive exercise. Thus, for each of the six subgroups, five mice were needed. The total sample size of 30 mice provided 89% power to detect differences among the means compared to the alternative of equal means, with a significance level of α 0.05, using an F test. The calculation was based on a pilot study that determined the size of variation in the means as 4.11 and assumed a common standard deviation of 5 within each group. The sample size was determined using PASS 15.0.5.

### Statistical analysis

The Shapiro-Wilk test was conducted to assess the normality for continuous variables. To examine the differences in body weight over time among the groups, we employed repeated measures analysis of variance (ANOVA). In the subsequent analyses, normally distributed variables were assessed using ANOVA, followed by a 2-sample t-test for each pair with Bonferroni correction. For non-normally distributed variables, we employed the Kruskal-Wallis test, followed by the Wilcoxon rank-sum test for each pair with Bonferroni correction. The data were analyzed using SPSS Statistics version 25, and the threshold for statistical significance was set at *P* < 0.05 (two-sided).

## Results

### Aerobic exercise represses the progressive development of obesity

**Figure 2:**
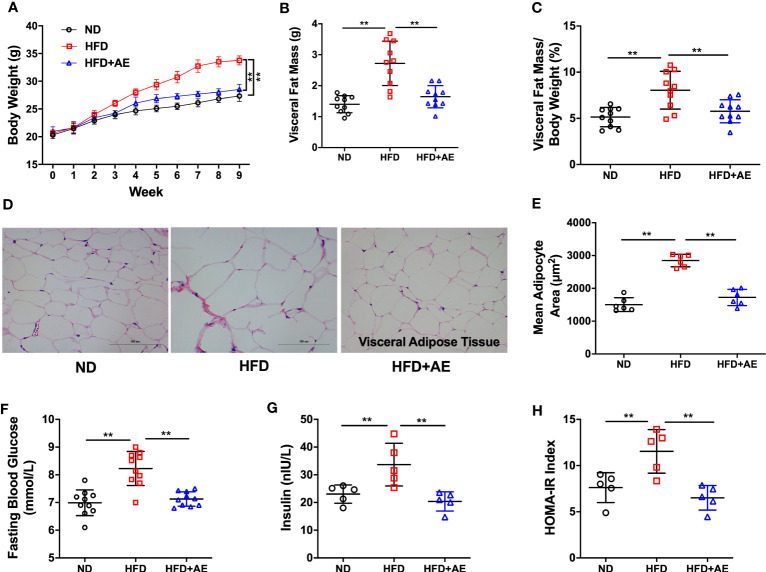
Eight weeks of aerobic exercise mitigates high-fat diet-induced obesity, high fasting blood glucose, and insulin resistance. **(A)** Changes in body weight, **(B)** Visceral fat mass, and **(C)** Visceral fat mass/body weight ratio (n=10 per group). **(D)** Representative histological images of visceral adipose tissue following hematoxylin and eosin staining and **(E)** Mean adipocyte area (n=5 per group). **(F)** Fasting blood glucose (n=10 per group), **(G)** insulin (n=5 per group), and **(H)** HOMA-IR (n=5 per group). ND, normal diet; HFD, high-fat diet; AE, aerobic exercise; HOMA-IR, homeostasis model assessment for insulin resistance. Data are reported as the mean ± standard deviation (SD). **p<0.01.

Mice fed an HFD displayed a significant increase in body weight (**Figure 2A**), visceral fat mass (**Figure 2B**), and visceral fat mass/body weight ratio (**Figure 2C**), as well as increased adipocyte hypertrophy (**Figures 2D, E**), a typical obesity phenotype, compared to those fed a normal diet. The HFD-induced obese phenotype was significantly attenuated by AE (**Figures 2A-E**). Additionally, HFD-fed mice presented significantly higher serum FBG, insulin, and HOMA-IR levels than the mice in the control group, and these obesity phenotypes were also significantly attenuated by AE (**Figures 2F-H**).

### Aerobic exercise attenuates fatal stress-induced cardiac dysfunction and myocardial injury in obese mice

**Figure 3:**
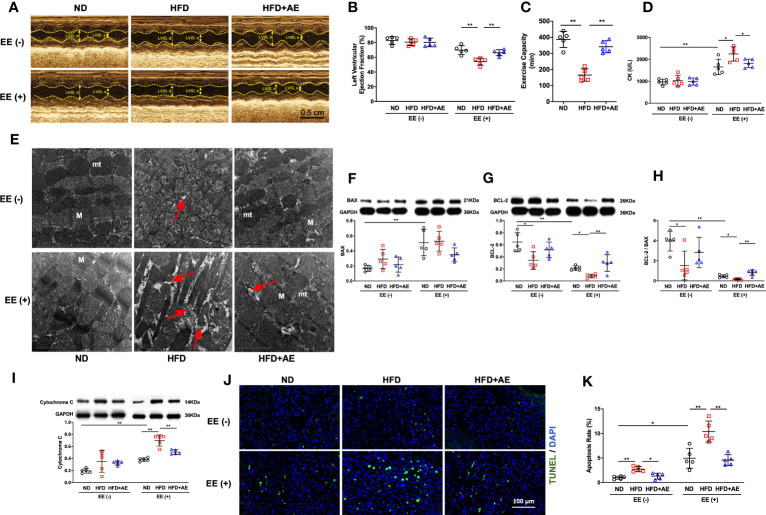
Aerobic exercise attenuates exhaustive exercise-induced myocardial injury in obese mice. **(A)** Representative M‐mode images of echocardiography in each treatment group. **(B)** Measurement of left ventricular ejection fraction by echocardiography. **(C)** Exercise capacity expressed as the swimming duration until exhaustion. **(D)** Serum CK concentration in obese mice with or without fatal stress stimulus. **(E)** Representative transmission electron microscopy images of the left ventricular myocardium. Damaged mitochondria, evidenced by mitochondrial swelling and the absence of cristae, and the rupture and deformation of myofibers (red arrows) are shown in the images. mt, mitochondria; M, myofiber; the scale bar = 2 μm. **(F)** BAX and **(G)** BCL-2 protein levels. **(H)** The BCL-2/BAX ratio. **(I)** Cytochrome C protein level. **(J)** Representative images and quantification of TUNEL-positive neurons. **(K)** Apoptosis rate. ND, normal diet; HFD, high-fat-diet; AE, aerobic exercise; LVID, left ventricular internal dimension; CK, creatine kinase; BCL-2, B-cell lymphoma-2; BAX, BCL-2-associated X protein. Data are reported as the mean ± standard deviation (SD) (n=5 per group). *p<0.05; **p<0.01.

The EE was employed to induce fatal stress in mice (12, 20), and resulted in a significant reduction in LVEF, indicating impaired cardiac function. In addition, obesity exacerbated the decrease in LVEF, whereas AE preconditioning significantly mitigated this exacerbation (**Figures 3A, B**). Furthermore, we observed that obesity impaired exercise capacity, as evaluated by the swimming duration until exhaustion (**Figure 3C**). These findings indicate that AE preconditioning effectively alleviated fatal stress-induced impairments in cardiac function and overall function.

CK is an enzyme located in the skeletal and heart muscles that leak into the bloodstream when these muscles are damaged, and elevated CK levels indicate a high probability of acute myocardial damage in patients with chest pain (27). We observed an increase in serum CK levels using an ELISA (**Figure 3D**) and detected severe myocardial mitochondria and sarcomere damage using transmission electron microscopy (**Figure 3E**), indicating that exhaustive exercise-induced fatal stress resulted in myocardial damage.

The life-or-death decision for a cell is mainly determined by the interaction between BCL-2 family members. Among these, BCL-2 and BAX are the main pro-survival and pro-apoptotic mediators, respectively (28). Cytochrome c presents in the mitochondrial intermembrane and intercristae space in healthy conditions, but leaks from damaged mitochondria into the cytosol to activate the apoptotic signaling pathway (29). Overexpression of BCL-2 blocks cytochrome c release from the mitochondria (30) whereas BAX promotes cytochrome c leakage through BAX oligomeric pores (31). We detected elevated cytochrome c and BAX protein levels, decreased BCL-2 levels, and a decline in the ratio of BCL-2 to BAX upon exhaustive exercise (**Figures 3F-I**). These changes were accompanied by an increase in apoptosis, as evidenced by an increase in the percentage of TUNEL-positive cells (**Figures 3J, K**).

Together, these results indicate that obesity impaired cardiac and overall function, and exacerbated fatal stress-induced myocardial injury and apoptosis. AE preconditioning significantly alleviated fatal stress-induced cardiac injury and apoptosis and reversed the exacerbation caused by the HFD.

### Aerobic exercise counteracts fatal stress-induced deficit in myocardial mitochondrial quality control in obese mice

**Figure 4:**
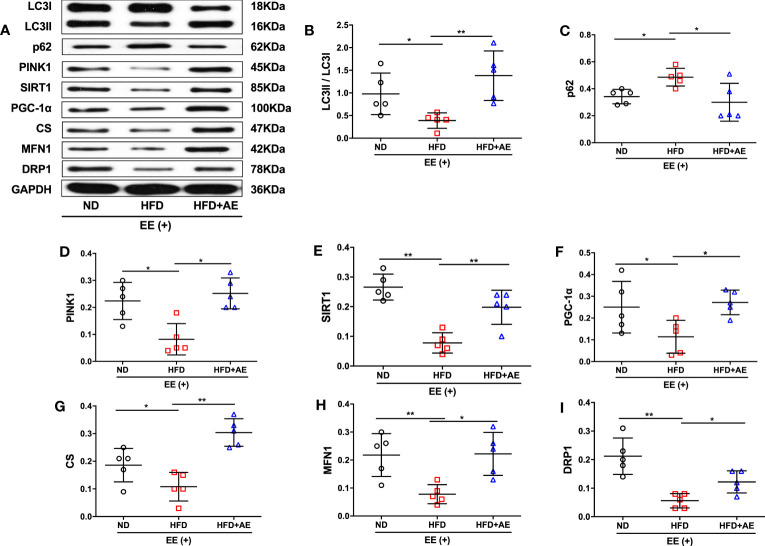
Aerobic exercise counteracts exhaustive exercise-induced deficit in myocardial mitochondrial quality control in obese mice. **(A)** Representative western blot images. Targeted protein gels are presented by lining up with the GAPDH gel, but analysed with its loading controls, respectively. **(B-I)** Levels of myocardial LC3 **(B)**, p62 **(C)**, PINK1 **(D)**, SIRT1 **(E)**, PGC-1α **(F)**, CS **(G)**, MFN1 **(H)**, and DRP1 **(I)**. ND, normal diet; HFD, high-fat diet; AE, aerobic exercise; EE, exhaustive exercise; LC3, microtubule-associated protein 1 light chain 3; PINK1, PTEN-induced putative kinase 1; SIRT1, Sirtuin-1; PGC-1α, peroxisome proliferator-activated receptor-γ coactivator-1α; CS, citrate synthase; MFN1, mitofusin 1; DRP1, dynamin-related protein 1. Data are reported as the mean ± standard deviation (SD) (n=5 per group). *p<0.05; **p<0.01.

The quality of mitochondria plays a crucial role in both apoptosis and survival (32). Elevation of DRP1 in mice after cardiac ischemia-reperfusion injury leads to an increase in mitochondrial fission (33), which may counteract damage by promoting the separation and removal of injured mitochondrial components (12). Additionally, obesity disorders can suppress MFN1-mediated mitochondrial fusion in the heart (34, 35) in rats, leading to increased cardiomyocyte apoptosis (35). Furthermore, knockout of the mitophagy regulator PINK1 has been reported to increase serum CK levels and susceptibility to cardiac injury in response to exhaustive exercise (19). Moreover, impaired activity of the mitochondrial biogenesis pathway, SIRT1/PGC-1α, and reduced mitochondrial volume in adipose tissue have been observed in obese children (36). Here, we observed a lower conversion from LC3-I to LC3-II, a standard marker for autophagosomes, accompanied by the suppression of PNIK1 and higher levels of p62 in the mice myocardium after exhaustive exercise, indicating that obesity significantly suppressed mitophagy in response to fatal stress (**Figures 4A-D**). AE preconditioning significantly reversed obesity-induced suppression of myocardial mitophagy in mice after exhaustive exercise (**Figures 4A-D**). In addition, obese mice that suffered exhaustive exercise presented a deficit in mitochondrial biogenesis, as evidenced by significantly lower protein expression of SIRT1 (**Figure 4E**), PGC-1α (**Figure 4F**), and CS (**Figure 4G**), a marker of mitochondrial volume, compared to mice fed a normal diet; these phenotypes were also significantly reversed by AE (**Figures 4E-G**). Moreover, in response to fatal stress, obese mice exhibited significantly lower protein levels of MFN1 (**Figure 4H**), a promoter of mitochondrial fusion, and DRP1 (**Figure 4I**), an activator of mitochondrial fission, compared to the mice in the normal diet group, indicating impaired myocardial mitochondrial fusion and fission. Similar to the other phenotypes, this impairment was diminished by AE preconditioning (**Figures 4H, I**).

### The effects of aerobic exercise on MIF/AMPK pathway

**Figure 5:**
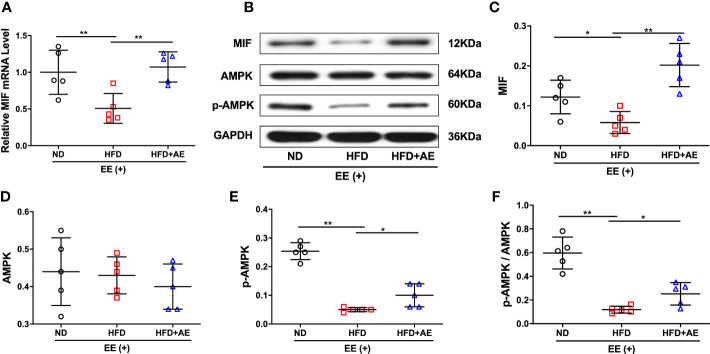
Effect of aerobic exercise on MIF/AMPK pathway activity in the myocardium of obese mice subjected to exhaustive exercise. **(A)** Reverse transcription-quantitative real-time PCR analysis of *MIF* mRNA level. **(B)** Representative western blot images. **(C-E)** Protein levels of MIF **(C)**, total AMPK **(D)**, and phosphorylated AMPK (pAMPK) **(E)**. **(F)** The ratio of pAMPK to total AMPK protein. ND, normal diet; HFD, high-fat diet; AE, aerobic exercise; EE, exhaustive exercise; MIF, macrophage inhibitory factor; AMPK, AMP-activated protein kinase. Data are reported as the mean ± standard deviation (SD) (n=5 per group). *p<0.05; **p<0.01.

A previous report by Meng et al. suggested that impaired myocardial MIF/AMPK activation exacerbates ischemia-reperfusion injury in HFD-induced obesity (37). AMPK is a key molecular transducer of exercise and regulator of MQC (38). In the present study, obesity significantly suppressed the MIF/AMPK pathway response to exhaustive exercise-induced fatal stress, as indicated by the lower levels of MIF mRNA and protein (**Figures 5A-C**), pAMPK, and the ratio of pAMPK/AMPK (**Figures 5D-F**) in the myocardium of mice after exhaustive exercise. These effects were significantly rescued by AE (**Figures 5A-F**).

## Discussion

Extreme stress is the most common cause of myocardial injury, followed by adverse cardiovascular events. In this study, we demonstrated that, in response to exhaustive exercise-induced fatal stress, obese mice sustained myocardial damage, which may involve a blunted MIF/AMPK pathway response, impaired MQC, increased release of cytochrome c from mitochondria, and increased apoptosis in the myocardium. Preconditioning with AE significantly reversed or ameliorated these changes. These results identified AE preconditioning as a valid cardioprotective intervention to prevent fatal stress-induced myocardial injury.

Compared to commonly used methods to establish myocardial damage in animal models, such as coronary ischemia-reperfusion and ligation of coronary artery, exhaustive exercise may better mimic stress in individuals with and without cardiovascular diseases (39, 40) and has been comprehensively applied in various *in vivo* studies (11, 13). In addition, our previous study showed that exhaustive exercise-induced fatal stress resulted in significant myocardial injury, as evidenced by increased serum CK levels and the broken ultrastructure of the myocardium in non-obese C57BL/6J male mice (12). These results suggested that exhaustive exercise can be used to elicit pathophysiological stress in animals.

A recent study by Li et al. reported that obesity spontaneously led to increased myocardial apoptosis accompanied by impaired cardiac structure and function in C57BLKS/J male mice (41). Our results indicate that obesity also impairs the ability of mice to protect the myocardium from fatal stress-induced damage. A growing number of clinical researches have reported that physical activity or exercise volume is significantly inversely associated with the risk of adverse cardiovascular events (42). In addition, several preclinical experiments suggest that exercise preconditioning can protect the myocardium from myocardial ischemia-reperfusion injury in non-obese mice (43, 44). In line with these studies, our previous study found that AE protects the myocardium from fatal stress-induced myocardial damage (12). Moreover, the anti-apoptotic effects of exercise, such as increasing the BCL-2/Bax ratio and reducing apoptosis after acute myocardial infarction in non-obese mice, have recently been reported (45). Building on these prior works, the present study demonstrated that regular AE can prevent fatal stress-induced myocardial apoptosis and damage in obese mice.

Exercise is considered a non-pharmacological tool for promoting mitochondrial health and resisting excessive apoptosis in multiple organs (46, 47) by regulating MQC, including mitochondrial biogenesis, fission and fusion, and mitophagy (48). Dysfunction in MQC is closely associated with obesity and an impaired response to stress. For example, after the knockout of β-cell-specific DRP1, a central regulator of mitochondrial fission, mice presented markedly abnormal mitochondrial morphology and impaired insulin secretion in response to high glucose stress (49). Furthermore, our previous study demonstrated that AE could upregulate myocardial mitophagy, and mitochondrial fusion and biogenesis in non-obese mice (12). This study showed that AE preconditioning regulates MQC in obese mice subjected to fatal stress, and, together with previous work, suggests that AE is an effective tool to rescue impaired MQC and improve the capacity in individuals with obesity to resist fatal stress-induced myocardial damage.

We also observed increased AMPK phosphorylation, which is consistent with improvement in MQC in the myocardium (38). AMPK phosphorylation has been shown to maintain mitochondrial fission at a relatively low but effective level by upregulating myocardial DRP1 expression, which resisted chronic Doxorubicin-induced increase of mitochondrial permeability transition pore and cell apoptosis (50). In addition, a 12-week exercise-induced increase in AMPK activation was reported to be associated with the recovery of mitochondrial fusion suppressed by doxorubicin, an AMPK inhibitor, in the myocardium (50). Furthermore, the initiation of mitophagy is dependent on AMPK activation, which promotes the removal of damaged mitochondrial and the reduction of cytochrome c release and ROS generation from mitochondria (38). Finally, Thr172 phosphorylation of the AMPK α subunit promotes mitochondrial biogenesis via SIRT1 activation. These data support a pivotal role of AMPK in regulating myocardial MQC in overcoming fatal stress-induced mitochondrial damage and cell apoptosis.

A study by Hyo Youl Moon et al. suggested that MIF involved the antidepressant effect of exercise (24). Additionally, previous studies have shown that MIF is an upstream regulator of AMPK and cardioprotection (51, 52); sustained exogenous administration of recombinant MIF efficiently activates AMPK phosphorylation and prevents hearts from ischemic injury (37, 53); MIF-engineered mesenchymal stem cells derived exosomes significantly activated the AKT signaling pathway, one of AMPK downstream pathways, and ameliorated cardiomyocyte apoptosis and cardiac dysfunction in rats with myocardial infarction (54). In this study, we detected increased MIF mRNA and protein levels in the myocardium of obese mice subjected to fatal stress, further supporting the MIF/AMPK pathway as a potential target for mitigating myocardial injury resulting from pathophysiological stress.

This preliminary study has several limitations. Firstly, it only observed the effects of an eight-week AE on cardiac function, myocardial structure, mitophagy, mitochondrial biogenesis, and dynamics in HFD-induced obese mice. Therefore, it cannot conclusively determine whether the improved quality of mitochondrial function contributes to AE-induced cardioprotection. Further research is necessary to explore the regulatory network among AE, mitochondrial quality, and cardioprotection in obesity models. Secondly, mitophagy is a dynamic process that progresses towards obesity and adapts to AE, hence it is better to assess the level of mitophagy activity by autophagic flux using chloroquine or Bafilomycin A1. Thirdly, this study only used male mice as an experimental model, which may limit the generalizability of the findings. Thus, future studies are encouraged to investigate the effects of varied exercise modalities on cardioprotection in both males and females. Furthermore, a growing body of research indicates that aerobic exercise-induced benefits may be linked to the reinforcement of antioxidant defenses in the body (55). However, in our study, we only observed the potential actions associated with the MIF/AMPK pathway, MQC, and apoptotic response in the myocardium. Consequently, we were unable to pinpoint the primary exercise responders responsible for protecting the myocardium from exhaustive exercise-induced damage. Encouraging future studies to investigate the redox mechanisms underlying aerobic exercise-induced myocardial protection would be beneficial.

## Conclusion

In this study, we provide evidence that the implementation of AE can prevent fatal stress-induced myocardial injury in obese mice, which offers new insights into regular AE as a precautionary measure for preventing adverse cardiovascular events in individuals with obesity.

## Data availability statement

The raw data supporting the conclusions of this article will be made available by the authors, without undue reservation.

## Ethics statement

The animal study was approved by Experimental Animal Welfare Ethics Committee of Central South University. The study was conducted in accordance with the local legislation and institutional requirements.

## Author contributions

YSD: Conceptualization, Methodology, Formal analysis, Writing-Original Draft, Funding acquisition. ZH: Methodology, Formal analysis, Writing-Original Draft, Visualization. BY: Methodology, Formal analysis, Writing - Review & Editing, Visualization. YD: Investigation, Methodology, Writing - Review & Editing, Visualization. LZ: Methodology, Writing - Review & Editing. YZ: Methodology, Writing - Review & Editing. YL: Methodology, Writing - Review & Editing. SW: Methodology, Writing - Review & Editing. NC: Methodology, Writing - Review & Editing. FY: Methodology, Writing - Review & Editing. SL: Funding acquisition, Supervision. All authors contributed to the article and approved the submitted version.

## References

[1] Timmis A Vardas P Townsend N Torbica A Katus H De Smedt D. European Society of Cardiology: cardiovascular disease statistics 2021. Eur Heart J (2022) 43(8):716–99. doi: 10.1093/eurheartj/ehab892 35016208

[2] Caleyachetty R Thomas GN Toulis KA Mohammed N Gokhale KM Balachandran K. Metabolically healthy obese and incident cardiovascular disease events among 3.5 million men and women. J Am Coll Cardiol (2017) 70(12):1429–37. doi: 10.1016/j.jacc.2017.07.763 28911506

[3] Ng ACT Delgado V Borlaug BA Bax JJ. Diabesity: the combined burden of obesity and diabetes on heart disease and the role of imaging. Nat Rev Cardiol (2021) 18(4):291–304. doi: 10.1038/s41569-020-00465-5 33188304

[4] Rayner JJ Peterzan MA Watson WD Clarke WT Neubauer S Rodgers CT. Myocardial energetics in obesity: enhanced atp delivery through creatine kinase with blunted stress response. Circulation (2020) 141(14):1152–63. doi: 10.1161/CIRCULATIONAHA.119.042770 PMC7144750 32138541

[5] Yu L Liang H Dong X Zhao G Jin Z Zhai M. Reduced silent information regulator 1 signaling exacerbates myocardial ischemia-reperfusion injury in type 2 diabetic rats and the protective effect of melatonin. J Pineal Res (2015) 59(3):376–90. doi: 10.1111/jpi.12269 26327197

[6] Feng R Cai M Wang X Zhang J Tian Z. Early aerobic exercise combined with hydrogen-rich saline as preconditioning protects myocardial injury induced by acute myocardial infarction in rats. Appl Biochem Biotechnol (2019) 187(3):663–76. doi: 10.1007/s12010-018-2841-0 30033489

[7] McGinnis GR Ballmann C Peters B Nanayakkara G Roberts M Amin R. Interleukin-6 mediates exercise preconditioning against myocardial ischemia reperfusion injury. Am J Physiol Heart Circulatory Physiol (2015) 308(11):H1423–H33. doi: 10.1152/ajpheart.00850.2014 25820396

[8] Mittleman MA Mostofsky E. Physical, psychological and chemical triggers of acute cardiovascular events: preventive strategies. Circulation (2011) 124(3):346–54. doi: 10.1161/CIRCULATIONAHA.110.968776 PMC3139921 21768552

[9] Song H Fang F Arnberg FK Mataix-Cols D Cruz LF Almqvist C. Stress related disorders and risk of cardiovascular disease: population based, sibling controlled cohort study. BMJ (2019) 365:l1255. doi: 10.1136/bmj.l1255 30971390 PMC6457109

[10] Zhang W Supervia M Dun Y Lennon RJ Ding R Sandhu G. The association between a second course of cardiac rehabilitation and cardiovascular outcomes following repeat percutaneous coronary intervention events. J Cardiopulmonary Rehabil Prev (2022) 43(2):101–8. doi: 10.1097/HCR.0000000000000717 35940745

[11] Xie M Jiang L Dun Y Zhang W Liu S. Trimetazidine combined with exercise improves exercise capacity and anti-fatal stress ability through enhancing mitochondrial quality control. Life Sci (2019) 224:157–68. doi: 10.1016/j.lfs.2019.03.027 30872179

[12] Dun Y Liu S Zhang W Xie M Qiu L. Exercise combined with Rhodiola Sacra supplementation improves exercise capacity and ameliorates exhaustive exercise-induced muscle damage through enhancement of mitochondrial quality control. Oxid Med Cell Longevity (2017) 2017:8024857. doi: 10.1155/2017/8024857 PMC5735688 29359009

[13] Jiang L Shen X Dun Y Xie M Fu S Zhang W. Exercise combined with trimetazidine improves anti-fatal stress capacity through enhancing autophagy and heat shock protein 70 of myocardium in mice. Int J Med Sci (2021) 18(7):1680–6. doi: 10.7150/ijms.53899 PMC7976563 33746584

[14] Vargas-Mendoza N Morales-Gonzalez A Madrigal-Santillan EO Madrigal-Bujaidar E Alvarez-Gonzalez I Garcia-Melo LF. Antioxidant and adaptative response mediated by nrf2 during physical exercise. Antioxidants (Basel) (2019) 8(6):196. doi: 10.3390/antiox8060196 31242588 PMC6617290

[15] Done AJ Traustadottir T. Nrf2 mediates redox adaptations to exercise. Redox Biol (2016) 10:191–9. doi: 10.1016/j.redox.2016.10.003 PMC5078682 27770706

[16] Hood DA Memme JM Oliveira AN Triolo M. Maintenance of skeletal muscle mitochondria in health, exercise, and aging. Annu Rev Physiol (2019) 81:19–41. doi: 10.1146/annurev-physiol-020518-114310 30216742

[17] Shutt TE McBride HM. Staying cool in difficult times: mitochondrial dynamics, quality control and the stress response. Biochim Biophys Acta (2013) 1833(2):417–24. doi: 10.1016/j.bbamcr.2012.05.024 22683990

[18] Roca-Portoles A Tait SWG. Mitochondrial quality control: from molecule to organelle. Cell Mol Life Sci (2021) 78(8):3853–66. doi: 10.1007/s00018-021-03775-0 PMC8106605 33782711

[19] Sliter DA Martinez J Hao L Chen X Sun N Fischer TD. Parkin and PINK1 mitigate STING-induced inflammation. Nature (2018) 561(7722):258–62. doi: 10.1038/s41586-018-0448-9 PMC7362342 30135585

[20] You B Dun Y Fu S Qi D Zhang W Liu Y. The treatment of Rhodiola mimics exercise to resist high-fat diet-induced muscle dysfunction via sirtuin1-dependent mechanisms. Front Pharmacol (2021) 12:646489. doi: 10.3389/fphar.2021.646489 33935745 PMC8082455

[21] Herzig S Shaw RJ. AMPK: guardian of metabolism and mitochondrial homeostasis. Nat Rev Mol Cell Biol (2018) 19(2):121–35. doi: 10.1038/nrm.2017.95 PMC5780224 28974774

[22] Li H Dun Y Zhang W You B Liu Y Fu S. Exercise improves lipid droplet metabolism disorder through activation of AMPK-mediated lipophagy in NAFLD. Life Sci (2021) 273:119314. doi: 10.1016/j.lfs.2021.119314 33667513

[23] Ma H Wang J Thomas DP Tong C Leng L Wang W. Impaired macrophage migration inhibitory factor-AMP-activated protein kinase activation and ischemic recovery in the senescent heart. Circulation (2010) 122(3):282–92. doi: 10.1161/CIRCULATIONAHA.110.953208 PMC2907453 20606117

[24] Moon HY Kim SH Yang YR Song P Yu HS Park HG. Macrophage migration inhibitory factor mediates the antidepressant actions of voluntary exercise. Proc Natl Acad Sci United States America (2012) 109(32):13094–9. doi: 10.1073/pnas.1205535109 PMC3420190 22826223

[25] Poole DC Copp SW Colburn TD Craig JC Allen DL Sturek M. Guidelines for animal exercise and training protocols for cardiovascular studies. Am J Physiol Heart Circulatory Physiol (2020) 318(5):H1100–H38. doi: 10.1152/ajpheart.00697.2019 PMC7254566 32196357

[26] Palomaki VA Koivukangas V Merilainen S Lehenkari P Karttunen TJ. A straightforward method for adipocyte size and count analysis using open-source software qupath. Adipocyte (2022) 11(1):99–107. doi: 10.1080/21623945.2022.2027610 35094637 PMC8803053

[27] Lee TH Weisberg MC Cook EF Daley K Brand DA Goldman L. Evaluation of creatine kinase and creatine kinase-MB for diagnosing myocardial infarction. Clin impact Emergency room. Arch Internal Med (1987) 147(1):115–21. doi: 10.1001/archinte.1987.00370010113026 3800513

[28] Czabotar PE Lessene G Strasser A Adams JM. Control of apoptosis by the BCL-2 protein family: implications for physiology and therapy. Nat Rev Mol Cell Biol (2014) 15(1):49–63. doi: 10.1038/nrm3722 24355989

[29] Garrido C Galluzzi L Brunet M Puig PE Didelot C Kroemer G. Mechanisms of cytochrome c release from mitochondria. Cell Death Differentiation (2006) 13(9):1423–33. doi: 10.1038/sj.cdd.4401950 16676004

[30] Yang J Liu X Bhalla K Kim CN Ibrado AM Cai J. Prevention of apoptosis by Bcl-2: release of cytochrome c from mitochondria blocked. Science (1997) 275(5303):1129–32. doi: 10.1126/science.275.5303.1129 9027314

[31] Zhang M Zheng J Nussinov R Ma B. Release of cytochrome c from bax pores at the mitochondrial membrane. Sci Rep (2017) 7(1):2635. doi: 10.1038/s41598-017-02825-7 28572603 PMC5453941

[32] Sedlackova L Korolchuk VI. Mitochondrial quality control as a key determinant of cell survival. Biochim Biophys Acta Mol Cell Res (2019) 1866(4):575–87. doi: 10.1016/j.bbamcr.2018.12.012 30594496

[33] Jin Q Li R Hu N Xin T Zhu P Hu S. DUSP1 alleviates cardiac ischemia/reperfusion injury by suppressing the Mff-required mitochondrial fission and Bnip3-related mitophagy via the JNK pathways. Redox Biol (2018) 14:576–87. doi: 10.1016/j.redox.2017.11.004 PMC5691221 29149759

[34] Chen D Li X Zhang L Zhu M Gao L. A high-fat diet impairs mitochondrial biogenesis, mitochondrial dynamics, and the respiratory chain complex in rat myocardial tissues. J Cell Biochem (2018) 119(11):9602. doi: 10.1002/jcb.27068 30171706 PMC6220867

[35] Ma T Huang X Zheng H Huang G Li W Liu X. SFRP2 improves mitochondrial dynamics and mitochondrial biogenesis, oxidative stress, and apoptosis in diabetic cardiomyopathy. Oxid Med Cell Longevity (2021) 2021:9265016. doi: 10.1155/2021/9265016 PMC8592716 34790288

[36] Zamora-Mendoza R Rosas-Vargas H Ramos-Cervantes MT Garcia-Zuniga P Perez-Lorenzana H Mendoza-Lorenzo P. Dysregulation of mitochondrial function and biogenesis modulators in adipose tissue of obese children. Int J Obes (2005) (2018) 42(4):618–24. doi: 10.1038/ijo.2017.274 29158541

[37] Meng F Li D Song B Li L. Impaired myocardial mif/ampk activation aggravates myocardial ischemia reperfusion injury in high-fat diet-induced obesity. Endocrine Metab Immune Disord Drug Targets (2019) 19(7):1046–54. doi: 10.2174/1871530319666190326143254 30914037

[38] Drake JC Wilson RJ Laker RC Guan Y Spaulding HR Nichenko AS. Mitochondria-localized AMPK responds to local energetics and contributes to exercise and energetic stress-induced mitophagy. Proc Natl Acad Sci United States America (2021) 118(37):e2025932118. doi: 10.1073/pnas.2025932118 PMC8449344 34493662

[39] Thompson PD Franklin BA Balady GJ Blair SN Corrado D Estes NA 3rd. Exercise and acute cardiovascular events placing the risks into perspective: a scientific statement from the American Heart Association Council on Nutrition, Physical Activity, and Metabolism and the Council on Clinical Cardiology. Circulation (2007) 115(17):2358–68. doi: 10.1161/CIRCULATIONAHA.107.181485 17468391

[40] Tofler GH Muller JE. Triggering of acute cardiovascular disease and potential preventive strategies. Circulation (2006) 114(17):1863–72. doi: 10.1161/CIRCULATIONAHA.105.596189 17060396

[41] Li X Wu Y Zhao J Wang H Tan J Yang M. Distinct cardiac energy metabolism and oxidative stress adaptations between obese and non-obese type 2 diabetes mellitus. Theranostics (2020) 10(6):2675–95. doi: 10.7150/thno.40735 PMC7052888 32194828

[42] Del Pozo Cruz B Ahmadi MN Lee IM Stamatakis E. Prospective associations of daily step counts and intensity with cancer and cardiovascular disease incidence and mortality and all-cause mortality. JAMA Internal Med (2022) 182(11):1139–48. doi: 10.1001/jamainternmed.2022.4000 PMC9468953 36094529

[43] Boardman NT Hafstad AD Lund J Rossvoll L Aasum E. Exercise of obese mice induces cardioprotection and oxygen sparing in hearts exposed to high-fat load. Am J Physiol Heart Circulatory Physiol (2017) 313(5):H1054–62. doi: 10.1152/ajpheart.00382.2017 28801525

[44] Hou Z Qin X Hu Y Zhang X Li G Wu J. Longterm exercise-derived exosomal mir-342-5p: a novel exerkine for cardioprotection. Circ Res (2019) 124(9):1386–400. doi: 10.1161/CIRCRESAHA.118.314635 30879399

[45] Tao L Bei Y Lin S Zhang H Zhou Y Jiang J. Exercise training protects against acute myocardial infarction via improving myocardial energy metabolism and mitochondrial biogenesis. Cell Physiol Biochem (2015) 37(1):162–75. doi: 10.1159/000430342 26303678

[46] Gomez-Cabrera MC Salvador-Pascual A Cabo H Ferrando B Viña J. Redox modulation of mitochondriogenesis in exercise. Does antioxidant supplementation blunt the benefits of exercise training? Free Radical Biol Med (2015) 86:37–46. doi: 10.1016/j.freeradbiomed.2015.04.006 25889822

[47] Gomez-Cabrera MC Viña J Olaso-Gonzalez G. Special issue: Exercise redox biology from health to performance. Redox Biol (2020) 35:101584. doi: 10.1016/j.redox.2020.101584 32448750 PMC7284920

[48] Sorriento D Di Vaia E Iaccarino G. Physical Exercise: A novel tool to protect mitochondrial health. Front Physiol (2021) 12:660068. doi: 10.3389/fphys.2021.660068 33986694 PMC8110831

[49] Hennings TG Chopra DG DeLeon ER VanDeusen HR Sesaki H Merrins MJ. *In vivo* deletion of beta-cell drp1 impairs insulin secretion without affecting islet oxygen consumption. Endocrinology (2018) 159(9):3245–56. doi: 10.1210/en.2018-00445 PMC6107751 30052866

[50] Marques-Aleixo I Santos-Alves E Torrella JR Oliveira PJ Magalhães J Ascensão A. Exercise and doxorubicin treatment modulate cardiac mitochondrial quality control signaling. Cardiovasc Toxicol (2018) 18(1):43–55. doi: 10.1007/s12012-017-9412-4 28536949

[51] Qi D Hu X Wu X Merk M Leng L Bucala R. Cardiac macrophage migration inhibitory factor inhibits JNK pathway activation and injury during ischemia/reperfusion. J Clin Invest (2009) 119(12):3807–16. doi: 10.1172/JCI39738 PMC2786800 19920350

[52] Ruze A Chen BD Liu F Chen XC Gai MT Li XM. Macrophage migration inhibitory factor plays an essential role in ischemic preconditioning-mediated cardioprotection. Clin Sci (London Engl 1979) (2019) 133(5):665–80. doi: 10.1042/CS20181013 30804219

[53] Wang J Tong C Yan X Yeung E Gandavadi S Hare AA. Limiting cardiac ischemic injury by pharmacological augmentation of macrophage migration inhibitory factor-AMP-activated protein kinase signal transduction. Circulation (2013) 128(3):225–36. doi: 10.1161/CIRCULATIONAHA.112.000862 PMC3781594 23753877

[54] Zhu W Sun L Zhao P Liu Y Zhang J Zhang Y. Macrophage migration inhibitory factor facilitates the therapeutic efficacy of mesenchymal stem cells derived exosomes in acute myocardial infarction through upregulating miR-133a-3p. J Nanobiotechnology (2021) 19(1):61. doi: 10.1186/s12951-021-00808-5 33639970 PMC7916292

[55] He F Li J Liu Z Chuang CC Yang W Zuo L. Redox mechanism of reactive oxygen species in exercise. Front Physiol (2016) 7:486. doi: 10.3389/fphys.2016.00486 27872595 PMC5097959

